# Experimental and Clinical Approaches to Preventing Aminoglycoside-Induced Ototoxicity: A Scoping Review

**DOI:** 10.3390/antiox14121467

**Published:** 2025-12-07

**Authors:** Marek Zadrożniak, Maciej Biskupski, Marcin Szymański, Jarogniew J. Łuszczki

**Affiliations:** 1 Department of Otolaryngology, Head and Neck Surgery, Medical University of Lublin, 20-090 Lublin, Poland; marek.zadrozniak@umlub.edu.pl (M.Z.); marcin.szymanski@umlub.edu.pl (M.S.); 2 Department of Plastic, Reconstructive Surgery and Microsurgery, Medical University of Lublin, 20-090 Lublin, Poland; maciej.biskupski@umlub.edu.pl; 3 Department of Occupational Medicine, Medical University of Lublin, 20-090 Lublin, Poland

**Keywords:** aminoglycosides, ototoxicity, prevention, hearing loss

## Abstract

(1) Aminoglycosides remain indispensable in modern medicine but share a serious dose-limiting adverse effect: irreversible cochleovestibular ototoxicity. (2) This scoping review systematically maps experimental and clinical strategies aimed at preventing aminoglycoside-induced hearing loss, integrating mechanistic insights across preclinical and translational domains. (3) Preclinical evidence, encompassing in vitro and in vivo studies, delineates three principal mechanistic ways of protection: (A) antioxidant and redox modulation, including N-acetyl-L-cysteine (NAC), vitamin C, edaravone, and selected phytochemicals, which counteract reactive oxygen species-mediated hair cell apoptosis; (B) mitochondrial stabilization with compounds such as mitoquinone, celastrol, and histone deacetylase inhibitors restoring bioenergetic and proteostatic balance; and (C) restriction of aminoglycoside entry through partial blockade of the mechano-electrical transduction channel, notably by ORC-13661 and related modulators. Additional strategies involve nitric oxide modulation, vasodilatory agents, and iron chelation. Efficacy, however, remains compound- and antibiotic-specific, with paradoxical effects observed for several drugs. Clinical evidence remains limited and methodologically diverse. Of the investigated pharmacologic interventions, aspirin provides the most robust and reproducible evidence of protection against gentamicin-induced hearing loss, whereas NAC demonstrates a consistent, but population-specific benefit among dialysis patients. In contrast, vitamin E—despite promising experimental findings—has failed to show clinically significant otoprotective effects in randomized human studies. (4) In conclusion, while experimental data establish a strong mechanistic basis for pharmacologic otoprotection, clinical studies remain few, underpowered, and methodologically inconsistent. Standardized, adequately powered, and mechanistically informed clinical trials are urgently needed to translate experimental promise into actionable otoprotective strategies.

## 1. Introduction

Aminoglycosides are rapidly bactericidal antibiotics characterized by amino-sugar structures and a specific mechanism of action—high-affinity binding to the A-site of 16S rRNA within the 30S ribosomal subunit, leading to impaired decoding, mistranslation, and eventual bacterial death [1]. Since the introduction of streptomycin in 1944, successive natural and semi-synthetic derivatives—neomycin, kanamycin, gentamycin, netilmicin, tobramycin, and amikacin—have been employed to treat a wide spectrum of serious bacterial infections. They exhibit potent activity against aerobic Gram-negative bacilli (notably *Enterobacteriaceae* and *Pseudomonas aeruginosa*), retain partial efficacy against Gram-positive pathogens such as *Staphylococcus aureus*, and remain useful for selected mycobacterial and zoonotic diseases, including *Yersinia pestis* and *Francisella tularensis* [2,3,4,5,6]. Their rapid, concentration-dependent killing kinetics, prolonged post-antibiotic effect, and synergistic interaction with β-lactam antibiotics have made them indispensable in empirical and targeted regimens for sepsis, intra-abdominal and respiratory infections, and as core components in the management of multidrug-resistant infections and mycobacterial diseases [1].

Aminoglycosides, despite their proven clinical utility, produce a major and potentially irreversible adverse effect: cochleovestibular ototoxicity. Aminoglycosides cross the blood–labyrinth barrier, likely through transporter- or transcytosis-mediated routes across the stria vascularis, and accumulate in perilymph before diffusing into the endolymph. Within the cochlea, they enter sensory hair cells predominantly via mechano-electrical transduction channels containing TMC1 subunits [7]. Once internalized, aminoglycosides induce phosphatidylinositol-4,5-bisphosphate depletion, K^+^ channel blockade, and excessive reactive oxygen species (ROS) generation, culminating in apoptotic hair cell death. The extent of ototoxic injury correlates with cumulative dose, exposure duration, and host susceptibility factors such as mitochondrial 12S rRNA mutations [8,9,10].

This scoping review aims to systematically map and synthesize the available experimental and clinical evidence on strategies to prevent aminoglycoside-induced cochleovestibular ototoxicity. By integrating mechanistic insights and therapeutic interventions across both preclinical and clinical domains, this review seeks to delineate the current knowledge landscape and identify key evidence gaps to guide future translational research.

## 2. Materials and Methods

**Figure 1:**
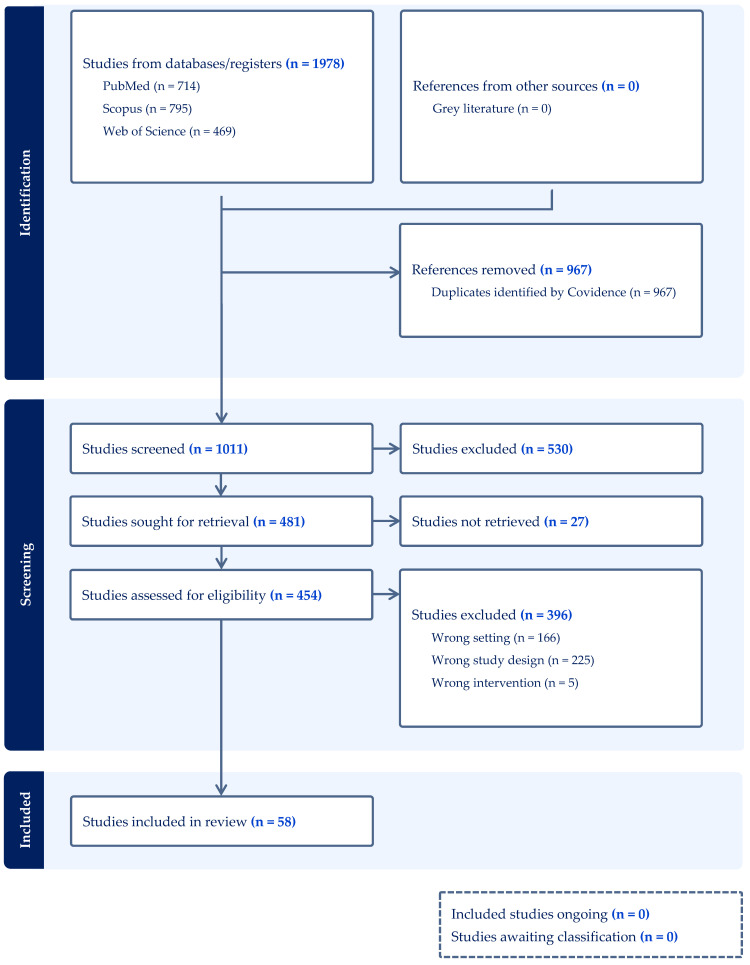
PRISMA 2020 flow diagram for systematic reviews.

This scoping review adheres to the Preferred Reporting Items for Systematic Reviews and Meta-Analyses (PRISMA) guidelines [11]. The PRISMA 2020 flowchart is presented in Figure 1. The article screening process was conducted using the Covidence systematic review software (Veritas Health Innovation, Melbourne, Australia; www.covidence.org, accessed on 2 November 2025). The literature searches were performed in databases such as PubMed, Web of Science, and Scopus. The literature search strategy was based on the following combination of keywords: (*aminoglycosides OR gentamicin OR kanamycin OR amikacin OR streptomycin OR neomycin*) *AND* (*ototoxicity OR otoprotection*). The search was limited to articles published in English. Included studies comprised only original research (observational, cohort, cross-sectional, and longitudinal studies). There were no restrictions on the year of publication.

## 3. Results

### 3.1. Experimental Evidence from in Vitro and in Vivo Studies

Across the experimental literature summarized in Table 1, putative otoprotective strategies against aminoglycoside-induced injury cluster around a limited number of mechanistic axes, the most consistent of which involves antioxidant and redox modulation. In both in vitro assays (most frequently HEI-OC1 auditory cell lines) and in vivo models (e.g., zebrafish, rats, and guinea pigs), the underlying pathobiology converges on a shared pattern of mitochondrial dysfunction, excess ROS formation, and activation of apoptotic and necrotic pathways following aminoglycoside entry into cochlear hair cells. Within this framework, compounds that restore intracellular thiols, scavenge free radicals, stabilize membranes, or support mitochondrial homeostasis frequently reduce hair cell loss or threshold shifts. Negative or paradoxical findings recur and protection is often drug-specific (gentamicin vs. amikacin vs. neomycin vs. kanamycin) and timing-dependent (pre- versus co-treatment).

Among classical antioxidants, N-acetyl-L-cysteine (NAC) remains the most extensively characterized. When aminoglycosides represent the principal insult, NAC consistently attenuates cochlear damage in vivo, demonstrating efficacy against amikacin alone and amikacin + furosemide co-exposure [12]. These observations are compatible with aminoglycoside-linked oxidative stress being a tractable target. Vitamin C likewise mitigated threshold shifts in amikacin + furosemide exposure [21], confirming a ROS-driven component of injury in conditions that enhance inner-ear drug penetration. In contrast, glutathione, despite strong biochemical plausibility, failed to protect in an otherwise identical amikacin + furosemide paradigm [20]. However, a study performed by Lautermann et al. showed that exogenous glutathione prevented gentamicin-related hearing loss only in nutrient-deficient animals [36], emphasizing that baseline metabolic reserve and cochlear redox capacity critically modulate response. These examples underscore that “antioxidant” is not a uniform pharmacologic category in the inner ear—pharmacokinetics, tissue entry, and intracellular targeting determine efficacy more than nominal antioxidant potency.

A broad group of membrane-stabilizing and polyphenolic antioxidants further supports this mechanism. α-Tocopherol (vitamin E) reduced gentamicin-induced hearing loss in guinea pigs [46], consistent with the inhibition of lipid peroxidation and preservation of hair cell membrane integrity. Other natural antioxidants demonstrated mixed results: garlic extract [37], silymarin [38], and pomegranate polyphenols [46] were protective against gentamicin, whereas 4-methylcatechol was not [38]. The ROS scavenger, edaravone, also attenuated gentamicin ototoxicity in vivo [39]. Ginkgo biloba, despite antioxidant reputation, paradoxically enhanced amikacin ototoxicity [16], perhaps due to hemodynamic effects in the cochlea. Flavonoid compounds such as baicalin [28], berberine chloride [13,65], and (−)-epigallocatechin-3-gallate [14] showed reproducible protective effects in vitro and in vivo through attenuation of ROS and partial restoration of mitochondrial function. Similarly, rutin [55] and cichoric acid [66]—both phenolic antioxidants—preserved hair cell survival in zebrafish models, acting through combined radical-scavenging and mitochondrial-support mechanisms.

Beyond antioxidant action, several agents exert protection through mitochondrial, endoplasmic reticulum, and epigenetic pathways. Mitoquinone, a mitochondria- targeted coenzyme Q derivative, protected against gentamicin, but not amikacin [15,31], highlights mechanistic heterogeneity among aminoglycosides. SkQR1, another mitochondria-directed antioxidant, and Bendavia (elamipretide) similarly preserved cochlear integrity under gentamicin exposure [33,50]. Upregulation of protein-folding and heat-shock responses also proved beneficial; celastrol, a natural HSF-1 inducer, consistently protected against gentamicin, kanamycin, and neomycin, even in knockout lines lacking HSP70 isoforms [47] and calreticulin, a Ca^2+^-buffering protein abundantly expressed in cochlear hair cells, bound gentamicin and sequestered it from pro-apoptotic targets such as CLIMP-63, thereby reducing free drug levels and mitigating cytotoxicity [32]. Epigenetic reprogramming further supports survival: suberoylanilide hydroxamic acid (SAHA), a histone deacetylase inhibitor, improved outcomes in kanamycin + furosemide models [61], and sodium butyrate promoted similar protection against gentamicin in vivo [53]. Additional modulators of mitochondrial bioenergetics include dihydronicotinamide riboside, which replenishes NAD (H) pools and attenuates kanamycin + furosemide damage [60], and pyrroloquinoline quinone, which scavenges ROS and activates mitochondrial biogenesis [30]. Collectively, these findings support the notion that enhancing mitochondrial resilience and stress-response signaling can blunt aminoglycoside toxicity, though efficacy remains compound- and antibiotic-specific.

A mechanistically direct and distinct approach involves restricting aminoglycoside entry into hair cells via the apical mechano-electrical transduction channel, the principal conduit for cationic drug uptake. ORC-13661 remains the most advanced compound in this class: it protects against gentamicin, amikacin, and neomycin in zebrafish and mammalian in vivo paradigms [19]. d-Tubocurarine and berbamine also reduced aminoglycoside injury through reversible mechano-electrical transduction-channel blockade, an effect corroborated in vivo [54]. Additional mechano-electrical transduction-pore or trafficking modulators identified—such as quinoxaline-5-carboxylic acid (Qx28)—also have been shown to reduce hair cell loss.

Other protective strategies modulate microcirculation, nitric oxide signaling, or osmotic stress, indirectly improving cochlear resilience. Mannitol acted both as an osmotic stabilizer and ROS scavenger against gentamicin injury [27]. Inhibitors of phosphodiesterase such as pentoxifylline and cilostazol conferred protection from amikacin in rats, likely through vasodilatory and antioxidant effects [17,18]. Nitric oxide modulation by N^G^-Nitro-L-arginine methyl ester (L-NAME) provided region-specific benefits, preferentially preserving the high-frequency (basal) cochlear regions where oxygen demand and aminoglycoside accumulation are the greatest [40].

A further subset of agents acts through anti-inflammatory, neuromodulatory, or metabolic signaling. Minocycline, with known anti-apoptotic and microglial-suppressive activity, limited gentamicin toxicity [23]. Brimonidine, an α_2_-adrenergic agonist, and telmisartan, an angiotensin AT_1_-receptor antagonist with peroxisome proliferator-activated receptor (PPAR)-γ agonist properties, similarly reduced gentamicin-induced damage in rats [24,25]. Pioglitazone and fenofibrate, both PPAR ligands, ameliorated gentamicin ototoxicity through improved mitochondrial and lipid metabolism [29,57]. Trimetazidine, by shifting cardiac-like metabolic fluxes toward glucose oxidation, and pasireotide, a somatostatin analog with anti-inflammatory effects, also demonstrated partial efficacy [35,56]. In kanamycin paradigms, rasagiline and selegiline, both monoamine oxidase (MAO)-B inhibitors, reduced cochlear injury [62,64], by decreasing ROS derived from monoamine oxidation and upregulating pro-survival signaling. Botanical preparations including *Salvia miltiorrhiza* [44] and the flavonoid fraction from *Drynaria fortunei* [43], further supported cochlear protection via antioxidative and anti-inflammatory pathways.

In parallel, iron-chelating strategies aim to suppress ROS generation and to reduce the formation of aminoglycoside–iron complexes that potentiate intracellular oxidative injury. Deferoxamine reduced gentamicin-induced damage in rats [22], while 2,3-dihydroxybenzoic acid, a salicylate derivative capable of Fe^3+^/Fe^2+^ sequestration, protected against kanamycin toxicity [63].

When examined by antibiotic class, efficacy patterns diverge. Gentamicin exhibits the broadest preclinical evidence base, with reproducible protection across antioxidant, mitochondrial, and mechano-electrical transduction-blocking agents. Amikacin follows, with consistent benefit from NAC [12], (−)-epigallocatechin-3-gallate [14], vitamin C [21], methionine [20], pentoxifylline [17], cilostazol [18], ORC-13661 [19], and berberine [13], but limited response to mitoquinone [15] and gluthathione [20]. Neomycin models confirm robust activity of celastrol [47], berberine [65], bizbenzoquinoline derivatives [42], ORC-13661 [19], Qx28 [48], cichoric acid [66], emricasan [67], d-tubocurarine, and berbamine [54], whereas sodium thiosulfate [67] and salicylate [26] remain ineffective. Kanamycin toxicity is mitigated by celastrol [47], rasagiline [64], selegiline [62], berberine chloride [13], 2,3-dihydroxybenzoic acid [63], and in combined injury paradigms by dihydronicotinamide riboside [60] and SAHA [61]. Among all in vitro and in vivo studies reviewed, tetramethylpyrazine is the only compound investigated for its protective effects specifically against streptomycin-induced ototoxicity (otoprotective action was linked to the attenuation of cellular stress responses and apoptosis, evidenced by reduced HSP70 and caspase-3 activation) [68].

**Figure 2:**
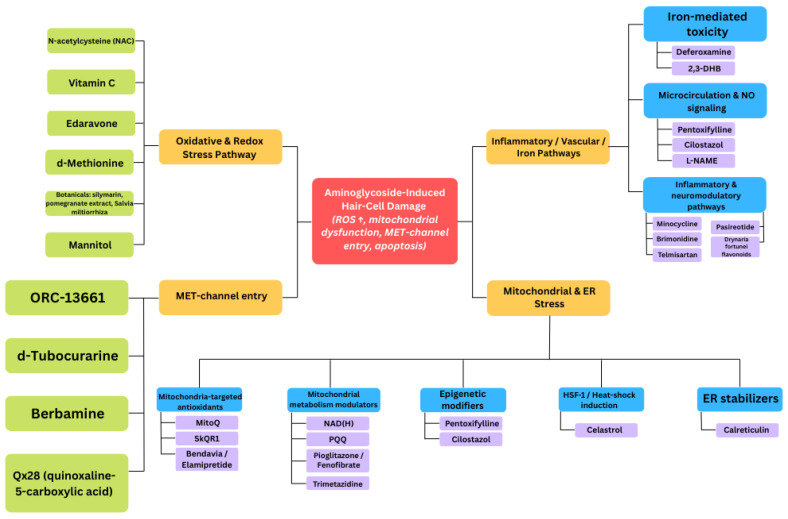
Schematic diagram illustrating potential mechanisms of protection against aminoglycoside-induced hair cell damage.

Taken together, preclinical evidence delineates three major mechanistic themes of protection: (1) attenuation of oxidative and inflammatory stress, (2) stabilization of mitochondrial and endoplasmic reticulum homeostasis, and (3) restriction of aminoglycoside entry through mechano-electrical transduction-pore modulation (Figure 2).

Despite extensive proof-of-concept work, these findings are fragmented across models, species, and antibiotics, and only a fraction of compounds show reproducible efficacy in more than one paradigm. The dominance of small-scale, short-term studies—with variable dosing, timing, and endpoints—underscores a key translational gap: while numerous compounds protect hair cells under controlled laboratory conditions, systematic validation, pharmacokinetic profiling, and evaluation of functional hearing outcomes remain scarce. Preclinical data thus provide a rich, but heterogeneous foundation that identifies promising mechanistic avenues, yet highlights the need for standardized, comparative frameworks before clinical translation can be achieved.

### 3.2. Clinical Evidence: Interventional Studies and Practice Changes

Relative to the preclinical breadth, human studies are few, generally small, and concentrated in specific clinical niches. Most evaluate co-therapies administered alongside aminoglycosides, especially aspirin/salicylates and NAC. Audiovestibular outcomes include conventional pure-tone audiometry (standard and extended high-frequency ranges) and, in some dialysis cohorts, otoacoustic emissions (TEOAE/DPOAE). Importantly, the criteria used to define ototoxicity vary considerably across studies, encompassing different threshold shifts, frequency ranges, and timing of post-treatment assessments, which complicates direct comparison of outcomes and the interpretation of protective efficacy. Supplementary Table S1 provides a detailed summary of clinical studies investigating pharmacologic and procedural strategies aimed at preventing aminoglycoside-associated ototoxicity.

The most convincing randomized evidence for pharmacological co-therapy comes from Sha et al. [69]. In a double-blind, placebo-controlled randomized trial of 195 adults, aspirin 3 g/day for 14 days during gentamicin therapy lowered ototoxicity (≥15 dB at both 6 and 8 kHz) from 13% with placebo to 3% with aspirin [69]. A smaller double-blind randomized study (n = 60) using 1.5 g/day for 7 days likewise favored aspirin: clinically relevant shifts at 4–8 kHz were rarer in the aspirin arm, and mean post-trial access thresholds differed significantly at 2, 4, and 8 kHz [70]. The concordant direction across two randomized controlled trials supports the translational validity of redox-based protection observed preclinically, that salicylates can blunt high-frequency loss during gentamicin exposure. In contrast, a randomized double-blind study of vitamin E (2.8 g/day for 7 days; n = 52) did not reduce ototoxicity (≥15 dB criterion) [71], despite preclinical signals for α-tocopherol [46]. This divergence underscores the lesson from the experimental literature: antioxidant class effects do not guarantee clinical efficacy, and compound-specific pharmacokinetics and cochlear distribution likely determine clinical performance.

The other recurrent co-therapy in the human literature is NAC, studied predominantly in renal-replacement populations receiving aminoglycosides for infection—groups at high risk of ototoxicity because of altered pharmacokinetics and prolonged exposure. In hemodialysis patients treated with gentamicin, an open-label randomized study (n = 40) administering NAC 600 mg twice daily throughout antibiotic therapy reported a significant overall reduction in ototoxicity (60% in controls vs. 25% with NAC; *p* = 0.025) [72]. Benefits were already evident at ≈7 days and persisted at ≈42 days after therapy: at early follow-up, 55% of controls vs. 20% of NAC recipients met ototoxicity criteria; at late follow-up it was 55% vs. 10%. Bilateral involvement was less common with NAC (30% vs. 5%), and the largest between-group differences occurred at high frequencies (6–12 kHz), where mean threshold shifts were significantly smaller in the NAC arm at both early and late follow-up [72]. These data dovetail with preclinical results, showing early protection of basal-turn hair cells by redox intervention.

In peritoneal dialysis patients receiving amikacin for peritonitis, three studies align on the early benefit for NAC. A randomized open-label trial (n = 40) administering NAC 600 mg twice daily for 1 month found better post-trial access results at 1 month across most test frequencies and both ears (with one non-significant exception at 2 kHz in the left ear), but group differences were no longer significant at 12 months, suggesting attenuation of effects or convergence over time [73]. A randomized placebo-controlled study (n = 46) examined OAEs and showed that NAC 600 mg twice daily for 2 weeks preserved or improved cochlear outer-hair cell function: TEOAEs improved at 1.5 and 2 kHz at week 4 (*p* = 0.011 and *p* = 0.014) and DPOAEs increased at 1 kHz (*p* < 0.001) and 8 kHz (*p* = 0.018) at weeks 1 and 4 compared with the baseline [74]. Another open-label randomized study (n = 60) using the same two-week NAC regimen reported substantially fewer cases meeting ototoxicity criteria at both 8 days and 28 days, and markedly smaller high-frequency post-trial access shifts at 28 days (≈−6 dB with NAC vs. ≈+17 dB in controls; *p* < 0.001 across low- and high-frequency composites) [75]. Taken together, these dialysis-cohort trials support NAC’s early protective signal—especially at high frequencies—during aminoglycoside therapy. The durability of benefit beyond the first few months is less certain, and generalizability to non-dialysis populations remains to be established.

An alternative line of clinical research explores localized inner ear delivery techniques, in which otoprotection is embedded within the treatment approach itself rather than achieved through adjunctive pharmacotherapy. In Ménière’s disease, a small retrospective series compared direct intratympanic gentamicin injection with a selective-window technique that placed gentamicin toward the oval window while covering the round window with dexamethasone. The direct-injection approach worsened high-frequency thresholds (4–8 kHz) and increased caloric weakness, whereas the selective-window method preserved post-trial access at low and high frequencies [76]. Because the primary aim was to compare Ménière’s treatment methods, patients self-selected the procedure, and no blinding occurred; therefore, causal attribution of a steroid-mediated otoprotective effect is limited. Nevertheless, the authors suggest that the findings of this study may indicate a clinically relevant otoprotective effect of dexamethasone against gentamicin-induced toxicity.

Two limitations pervade this clinical corpus. First, scale and design: most studies are small, several are open-label, and many are restricted to dialysis (hemodialysis or CAPD peritonitis) or infant cardiac cohorts—settings with unique drug kinetics and comorbid risks that limit generalizability. Only the larger aspirin trial provides double-blind, placebo-controlled evidence in a broader adult population on gentamicin [70]. Second, heterogeneity in outcome definitions, test batteries, and follow-up schedules complicates cross-trial synthesis and precludes meta-analytic pooling on uniform endpoints. These features collectively define the translational gap: many agents show promise in vitro/in vivo, but few have been tested in humans, and existing trials are typically underpowered, heterogeneous, and population-restricted.

### 3.3. Future Directions

Bridging the gap between mechanistic insight and clinical application in otoprotection requires focused, translational research rather than further isolated preclinical work. Experimental data have clearly defined key injury pathways—oxidative stress, mitochondrial dysfunction, or drug entry through mechano-electrical transduction channels—but most protective compounds remain untested under clinically realistic conditions. A central priority is pharmacokinetic alignment between models and patients. Compounds such as ORC-13661, celastrol, or pentoxifylline should be evaluated in early phase human studies that quantify systemic and cochlear exposure, confirm achievable protective concentrations, and ensure no interference with antimicrobial efficacy. Since aminoglycosides and loop diuretics are often co-administered, new in vivo and clinical studies must reproduce this dual insult to evaluate synergistic toxicity and determine whether protective agents maintain efficacy under these conditions. Equally important is the standardization of outcome measures. Future trials should use consistent ototoxicity definitions and sensitive endpoints—extended high-frequency audiometry, otoacoustic emissions, or vestibular testing—to allow for meaningful comparison and data synthesis across studies. Finally, large multicenter pragmatic trials are needed to test validated interventions. As of the time of writing this review (October 2025), there are no registered clinical trials investigating preventive interventions for aminoglycoside-induced ototoxicity, in contrast there is a growing number of ongoing studies targeting cisplatin-related hearing loss, for instance (NAC, sodium thiosulfate, transtympanic Ringer’s lactate, and SENS-401) [77,78]. This absence underscores the need for translational initiatives that extend the momentum of oncology-driven otoprotection into infectious disease and nephrology settings. Overall, future research should move from mechanistic demonstration to translational validation—integrating pharmacokinetics, standardized monitoring, and real-world exposure models to achieve clinically actionable strategies for preventing drug-induced hearing loss.

## 4. Conclusions

This scoping review reveals a striking imbalance between extensive preclinical progress and the limited, fragmented clinical evidence on strategies to prevent aminoglycoside-induced ototoxicity. (1) A robust mechanistic foundation exists at the experimental level, highlighting three consistently protective pathways: antioxidant and redox modulation, mitochondrial stabilization, and restriction of aminoglycoside entry through the mechano-electrical transduction channel. Yet, protection is often compound-specific and context-dependent, with conflicting outcomes such as Ginkgo biloba worsening amikacin toxicity or mitoquinone protecting against gentamicin, but not amikacin, underscoring that a single universal otoprotective approach is unlikely to succeed across all agents and conditions. (2) Clinical evidence remains sparse and methodologically inconsistent. Only a handful of human studies—mostly small, single-center trials—suggest benefits from aspirin or NAC in gentamicin-treated or dialysis populations. Most translational work and nearly all intervention studies target aminoglycoside injury. Collectively, these findings emphasize that while the experimental landscape of otoprotection against aminoglycoside injury is scientifically rich, the clinical evidence base remains immature, fragmented, and urgently in need of standardization and expansion to achieve actionable, generalizable prevention strategies.

## Figures and Tables

**Table 1:** Summary of in vitro and in vivo studies evaluating potential otoprotective agents against aminoglycoside-induced ototoxicity.

Tested Compound	Aminoglycoside Antibiotic	Experimental Model	Potential Otoprotective Effect	Reference
N-acetyl-L-cysteine	Amikacin	Albino Swiss outbred mice	yes	[12]
Berberine chloride	Amikacin	Mice	yes	[13]
(−)-Epigallocatechin-3-gallate	Amikacin	Zebrafish AB wild-type	yes	[14]
Mitoquinone	Amikacin	Guinea pigs	no	[15]
N-acetyl-L-cysteine	Amikacin (+furosemide)	Albino Swiss outbred mice	yes	[12]
Ginkgo biloba	Amikacin	Sprague Dawley rats	no (enhance)	[16]
Pentoxifylline	amikacin	Wistar rats	yes	[17]
Cilostazol	amikacin	Wistar rats	yes	[18]
ORC-13661	amikacin	Zebrafish, CD-1 mice	yes	[19]
Methionine	amikacin (+furosemide)	Albino Swiss outbred mice	yes	[20]
Glutathione	amikacin (+furosemide)	Albino Swiss outbred mice	no	[20]
Vitamin C	amikacin (+furosemide)	Albino Swiss outbred mice	yes	[21]
Deferoxamine	gentamicin	Sprague Dawley rats	yes	[22]
Minocycline	gentamicin	Sprague Dawley rats	yes	[23]
Brimonidine	gentamicin	Wistar rat pups	yes	[24]
Telmisartan	gentamicin	Wistar rat pups	yes	[25]
Berberine chloride	gentamicin	Mice	yes	[13]
Salicylate	gentamicin	Wistar rat pups	yes	[26]
Mannitol	gentamicin	Rats	yes	[27]
Baicalin	gentamicin	HEI-OC1 cell line	yes	[28]
(−)-Epigallocatechin-3-gallate	gentamicin	Zebrafish AB wild-type	yes	[14]
Pioglitazone	gentamicin	C57BL/6N mouse pups	yes	[29]
Pyrroloquinoline quinone	gentamicin	HEI-OC1 cell line	yes	[30]
Mitoquinone	gentamicin	Guinea pigs	yes	[31]
Calreticulin	gentamicin	HEI-OC1 cells	yes	[32]
SkQR1	gentamicin	Rats	yes	[33]
Osthole	gentamicin	HEI-OC1 cell line, zebrafish	yes	[34]
Trimetazidine	gentamicin	Albino Swiss mice	yes	[35]
Glutathione	gentamicin	Guinea pigs	yes (only in animals in a state of nutrient deficiency)	[36]
Garlic	gentamicin	Wistar rats	yes	[37]
Silymarin	gentamicin	Guinea pigs	yes	[38]
4-Methylcatechol	gentamicin	Guinea pigs	no	[38]
Edaravone	gentamicin	guinea pigs	yes	[39]
Nitro-L-arginine methyl ester	gentamicin	Sprague Dawley rats	yes (only in high-frequency range)	[40]
Pomegranate	gentamicin	Wistar rats	yes	[41]
Bizbenzoquinoline	gentamicin	Zebrafish	yes	[42]
Flavanoid fraction from *Drynaria fortunei*	gentamicin	Guinea pigs	yes	[43]
*Salvia miltiorrhiza*	gentamicin	Guinea pigs	yes	[44]
(-)-Butaclamol	gentamicin	Zebrafish, HEI-OC1 line	yes	[45]
alpha-Tocopherol	gentamicin	Albino guinea pigs	yes	[46]
Celastrol	gentamicin	CBA/J, Hsf-1−/−, HSP70.1/3−/−mice	yes	[47]
Quinoxaline-5-carboxylic acid (Qx28)	gentamicin	Zebrafish	yes	[48]
Concanavalin A	gentamicin	Rats	yes	[49]
Bendavia	gentamicin	Zebrafish	yes	[50]
Peptide from *Netunea arthritica cumingii*	gentamicin	Zebrafish	yes	[51]
Adjudin	gentamicin	Sprague Dawley rats, C57BL/6J mice	yes	[52]
Sodium butyrate	gentamicin	Guinea pigs	yes	[53]
d-Tubocurarine and Berbamine	gentamicin	Zebrafish, CD-1 mice	yes	[54]
Rutin	gentamicin	Zebrafish	yes	[55]
Pasireotide	gentamicin	C57BL/6N mice	yes	[56]
Fenofibrate	gentamicin	Sprague Dawley rats	yes	[57]
Hangesha-shin-to (TJ-014)	gentamicin	Rats	yes	[58]
Taurine	gentamicin (+furosemide)	Guinea pigs	yes	[59]
ORC-13661	gentamicin (+furosemide)	Zebrafish, CD-1 mice	yes	[19]
Dihydronicotinamide riboside	kanamycin (+furosemide)	C57BL/6J mice	yes	[60]
Suberoylanilide hydroxamic acid (SAHA)	kanamycin (+furosemide)	FVB/NJ and C57BL/6J wild-type mice	yes	[61]
Berberine chloride	kanamycin	Mice	yes	[13]
Selegiline	kanamycin	CD-1 mice		[62]
Celastrol	kanamycin	CBA/J, Hsf-1−/−, HSP70.1/3−/−mice	yes	[47]
2,3-Dihydroxybenzoic acid	kanamycin	Albino mice of the strain Balb/cA	yes	[63]
Rasagiline	kanamycin	CD-1 male mice	yes	[64]
Salicylate	neomycin	Wistar rat pups	no	[26]
Celastrol	neomycin	CBA/J, Hsf-1−/−, HSP70.1/3−/−mice	yes	[47]
Berberine	neomycin	Mice	yes	[65]
Bizbenzoquinoline	neomycin	Zebrafish	yes	[42]
ORC-13661	neomycin	Zebrafish, CD-1 mice	yes	[19]
Quinoxaline-5-carboxylic acid (Qx28)	neomycin	Zebrafish	yes	[48]
Cichoric Acid	neomycin	Zebrafish	yes	[66]
Sodium thiosulfate	neomycin	HEI-OC1, phoenix auditory cells and primary rat SGN cultures	no	[67]
Emricasan	neomycin	HEI-OC1, phoenix auditory cells and primary rat SGN cultures	yes	[67]
d-Tubocurarine and Berbamine	neomycin	Zebrafish, CD-1 mice	yes	[54]
Tetramethylpyrazine	streptomycin	Guinea pigs	yes	[68]

## Data Availability

No new data were created or analyzed in this study. Data sharing is not applicable to this article.

## Supplementary Materials

The following supporting information can be downloaded at: https://www.mdpi.com/article/10.3390/antiox14121467/s1; Table S1: Clinical studies on prevention of aminoglycoside-associated ototoxicity.

## Abbreviations

The following abbreviations are used in this manuscript:

CAPD	Continuous ambulatory peritoneal dialysis
NAC	N-acetyl-L-cysteine
OAEs	Otoacoustic emissions
ROS	Reactive oxygen species
SAHA	Suberoylanilide hydroxamic acid
