# Supplementary material for: Experimental and Clinical Approaches to Preventing Aminoglycoside-Induced Ototoxicity: A Scoping Review
Source: Antioxidants (Basel). 2025 Dec 7;14(12):1467. doi: 10.3390/antiox14121467 (PMC12729955; doi:10.3390/antiox14121467)
Supplement: Supplementary file 1 [file antioxidants-14-01467-s001.zip › antioxidants-3997908-supplementary.pdf]

Review

Experimental and clinical approaches to preventing aminoglycoside–induced ototoxicity: a scoping review

Marek Zadrożniak [1], Maciej Biskupski [2], Marcin Szymański [1] and Jarogniew J. Łuszczki [3,*]

Supplementary Table 1. Clinical studies on prevention of aminoglycoside–associated ototoxicity.

Reference	Study design	Population	Ototoxic agent	Intervention	Audiovestibular Monitoring	Results	Key findings	Limitations
[69]	randomized double-blind placebo-controlled study	195 (106 in control group, 89 in aspirin group; 24 females and 171 males; average age - 25,6 years; average weight - 61,2 kg)	gentamicin (patients received 80 to 160 mg of gentamicin i.v. usually for 5 to 7 days; the mean total dose of gentamicin was 981 mg)	The intervention group received 14 days of supplementation with 3 g of aspirin daily, administered in three divided doses.	The hearing test was performed five to seven weeks after treatment, with ototoxicity defined as a ≥15 dB threshold shift from baseline at both 6 and 8 kHz, in either one or both ears.	In the placebo group, the occurrence of hearing loss was consistent with the expected rate (13%), whereas in the aspirin group it was markedly reduced to 3%.	The results suggest that vitamin C may exert an otoprotective effect against gentamicin-induced ototoxicity.	Significant predominance of men over women in both study groups.

[70]	randomized double-blind placebo-controlled study	60 (30 in intervention group, 30 in aspirin group; groups were "similar with respect to gender, age, and weight")	gentamicin (240 mg/day - 80 mg every 8 hours for 7 days)	The intervention group received 1.5 g of aspirin daily (500 mg/8 hours).	PTA (pure-tone audiometry) at 250, 500, 1000, 2000, 4000, 8000 Hz frequencies and speech discrimination testing were performed at baseline, on day 8, and again on day 15 of the study.	In the control group, clinically relevant threshold shifts (≥15 dB) were observed in 11 patients (36%) at 4000 Hz and in 6 patients (20%) at 8000 Hz, whereas in the aspirin group only a single case was recorded at each of these frequencies. Mean PTA thresholds showed significant differences at 2000, 4000, and 8000 Hz, with no significant changes at the other frequencies.	The results suggest that vitamin C may exert an otoprotective effect against gentamicin-induced ototoxicity.	Small number of study participants. At the beginning of the study except<br><br>in 2, 4, and 8 kHz frequencies<br><br>threshold of PTA in the aspirin group was a little higher<br><br>than the placebo group. There is no information on how many days aspirin was administered.
[71]	randomized double-blind placebo-controlled study	52 (29 in control group, 23 in intervention group; 28 females and 24 males; average age - 30,6 years)	gentamicin (240 mg/day - 80 mg every 8 hours for 7 days)	The intervention group received 7 days of supplementation with 2,8 g of vitamin E daily, administered in three divided into three portions of 1200, 800 and 800 mg.	PTA at 0,125 - 14 kHz frequencies was performed using Itera Sn 171803 Audiometer at baseline and at the follow-up visit 6 to 8 weeks after the therapy had finished. Ototoxicity was defined as a ≥15 dB threshold shift from baseline.	Two of 23 patients (8,7%) exhibited hearing loss in the intervention group, while three of 29 patients (10,3%) did sa in the control group.	Vitamin E has been not confirmed to possess any protective action against gentamicin-induced ototoxicity.	Small number of study participants.

[72]	randomized open-label controlled study	40 (20 in control group, 20 in intervention group; 15 females and 25 males; average age - 62,8 years; average weight - 73,5 kg); the study was conducted in patients undergoing hemodialysis.	gentamicin (patients received antibiotic therapy for an average duration of 14.5 days, with a cumulative antibiotic dose of 685 mg)	The intervention group received supplementation with N-acetylcysteine (NAC) 600 mg twice daily. NAC administration was initiated at enrollment and continued throughout gentamicin therapy until the first follow-up otologic examination, scheduled about a week after completion of antibiotic treatment.	PTA at 250, 500, 1000, 2000, 3000, 4000, 6000, 8000, and 12,000 Hz was performed using a GSI 16 audiometer. Follow-up otologic examinations were carried out 7 (early follow-up) and 42 days (late follow-up) after completing gentamicin therapy.	Ototoxicity occurred in 12 patients (60%) in the control group versus 5 patients (25%) in the NAC group, corresponding to a 41.6% risk reduction (p=0.025). At early follow-up (7±3 days post-therapy), ototoxicity was present in 11 controls (55%) and 4 NAC patients (20%, p=0.022). At late follow-up (42±3 days), 11 controls (55%) and 2 NAC patients (10%) still met ototoxicity criteria (p=0.002). Bilateral ototoxicity developed in 6 controls (30%) compared with 1 NAC patient (5%, p=0.037). The greatest differences were observed at high frequencies (6 000, 8 000, and 12 000 Hz): mean threshold shifts were 5.83±5.14 dB vs. 2.00±3.80 dB at early follow-up (p=0.011), and 7.00±8.15 dB vs. 2.08±5.18 dB at late follow-up (p=0.029).	NAC may be used as an effective, and safe drug for prevention of gentamicin-induced ototoxicity in hemodialysis patients.	Small number of study participants; the study was limited to patients undergoing hemodialysis.

[73]	randomized open-label controlled study	40 (17 in control group, 23 in intervention group; 15 females and 25 males; average age - 62,8 years; average BMI - 26,3 kg/m2); the study was conducted in patients undergoing continuous ambulatory peritoneal dialysis (CAPD) who developed peritonitis	amikacin (patients received 2 mg/kg daily of amikacin i.v. usually for 4 to 20 days; the mean total dose of amikacin was 1000 mg)	The intervention group received supplementation with N-acetylcysteine (NAC) 600 mg twice daily for 1 month.	PTA at 2000, 3000, 4000, 6000 Hz was performed using a AC 40 Audiometer. Hearing function tests were performed on the 2 groups before treatment (baseline), follow-up otologic examinations were carried out 1 month (early follow-up) and after 1 year (late follow-up) after starting treatment for peritonitis related to CAPD. Ototoxicity was defined as an increase in the auditory threshold by at least 20 dB at any 1 test frequency, or at least 10 dB at any 2 adjacent frequencies, or loss of response at 3 consecutive frequencies between the baseline and follow-up studies in the worse ear.	Patients taking NAC had better PTA results in both ears at 1 month except at 2,000 Hz for the left ear, which wasn't significantly different between the 2 groups. patients taking NAC had generally better PTA results at 12 months, differences between the 2 groups were not statistically different.	NAC may be otoprotective against amikacin-induced ototoxicity, although the effect does not appear to be long-lasting.	Small number of study participants; the study was limited to patients undergoing CAPD who developed peritonitis. Baseline white blood cell count was significantly higher in the NAC group than in the control group (p = 0.030). Patients taking NAC had worse baseline PTA results at 2,000 Hz for both the right and the left ears.
[74]	randomized placebo-controlled study	46 (23 in control group, 23 in intervention group; 23 females and 23 males; average	amikacin (patients received 2 mg/kg daily of amikacin i.v. usually for 4 to 21 days; the mean total dose of	The intervention group received supplementation with N-acetylcysteine (NAC) 600 mg twice	Audiovestibular monitoring included transient-evoked otoacoustic emissions (TEOAEs) and distortion product	Patients receiving NAC demonstrated preservation or improvement of otoacoustic emission responses. In the NAC	NAC is found to be safe and effective in amikacin-related ototoxicity in patients with PD-	Small number of study participants; the study was limited to patients undergoing

		age - 48,1 years; average BMI - 26,9 kg/m2); the study was conducted in patients undergoing continuous ambulatory peritoneal dialysis (CAPD) who developed peritonitis	amikacin was 1100 mg)	daily for 2 weeks.	otoacoustic emissions (DPOAEs), performed using a Madsen Capella Cochlear Emission Analyzer. Baseline measurements were obtained prior to amikacin therapy, with follow-up testing at 1 and 4 weeks. TEOAEs were recorded in fast-screen mode with 40 µs clicks at 75 dB SPL (rate 50/s, analysis 3–12.5 ms), and responses were evaluated at 1000, 1500, 2000, 3000, and 4000 Hz. DPOAEs were elicited with f2/f1 ratio of 1.21, L1/L2 intensities of 65/55 dB SPL, and responses were recorded at 500–8000 Hz; testing was terminated when S/N ratio reached 6 dB or after 1000 scans. The primary outcome was the signal-to-noise ratio (S/N), defined as the observed emission level minus the sum of subject noise and system distortion.	group, TEOAEs showed no decline at any frequency except 4000 Hz, with significant improvements at 1500 and 2000 Hz ($p=0.011$ and $p=0.014$). DPOAEs revealed significant increases at 1000 Hz ($p<0.001$) and 8000 Hz ($p=0.018$) compared with baseline.	related peritonitis.	CAPD who developed peritonitis.

[75]	randomized open-label controlled study	60 (30 in control group, 30 in intervention group; average age - 47,5 years; average BMI - 26,9 kg/m2); the study was conducted in patients undergoing continuous ambulatory peritoneal dialysis (CAPD) who developed peritonitis	amikacin (patients received 2 mg/kg daily of amikacin i.v. usually for 4 to 21 days; the mean total dose of amikacin was 1100 mg)	The intervention group received supplementation with N-acetylcysteine (NAC) 600 mg twice daily for 2 weeks during treatment for CAPD peritonitis.	PTA at 250, 500, 1000, 2000, 3000, 4000, 6000, 8000, 10000, 12000, 14000 and 16000 Hz was performed using a AC 40 Audiometer. Hearing function tests were performed on the two groups before treatment (baseline). Follow-up otologic examinations were carried out 8 days (early follow-up) and 28 days after starting treatment for CAPD peritonitis. Ototoxicity was defined as an increase in the auditory threshold by at least 20 dB at any one test frequency or at least 10 dB at any two adjacent frequencies or loss of response at three consecutive frequencies between the baseline and follow-up studies in the worse ear.	At early follow-up, ototoxicity was detected in 3 patients (10%) in the control group versus 1 patient (3.3%) in the NAC group at low frequencies (250-8000 Hz), and in 9 patients (30%) versus 1 patient (3.3%) at high frequencies (10000-16000 Hz). At late follow-up, 15 patients (50%) in the control group and 1 patient (3.3%) in the NAC group fulfilled ototoxicity criteria at low frequencies, while at high frequencies the corresponding rates were 21 patients (70%) versus 1 patient (3.3%). Mean threshold shifts also favored NAC: at early follow-up, at low frequencies hearing threshold changed by 3.16±6.25 dB in controls versus 1.36±5.85 dB in NAC patients ($p=0.25$), and at high frequencies by 5.43±8.56 dB versus −4.73±7.40 dB. At late follow-up, PTA at low	NAC may have a curative effect on impaired hearing functions in addition to a protective effect on high-frequency hearing functions.	Small number of study participants; the study was limited to patients undergoing CAPD who developed peritonitis

						frequencies deteriorated by 12.76±6.22 dB in controls compared with −1.86±5.98 dB in NAC patients (p<0.001), while at high frequencies worsened by 16.93±8.10 dB in controls compared with −5.96±8.89 dB in the NAC group (p<0.001).		
[76]	retrospective case review study (two different methods of intratympanic (IT) gentamicin administration in Meniere's Disease were evaluated - direct injection vs. selective window procedure using gel foam soaked in dexamethasone)	25 (15 patients received direct IT gentamicin injection and 10 underwent the selective window procedure.; 13 females and 12 males; average age - 47,9 years)	gentamicin (IT injection: 30.7 mg/mL, 1 mL weekly up to 3 doses; selective window: gentamicin 4 mg/mL applied via gel foam to oval window with dexamethasone on round window)	-	PTA at 250, 500, 1000, 2000, 4000, 6000, 8000 Hz bithermal caloric testing were performed. Baseline and post-treatment assessments - mean follow-up 17–19 months.	Direct IT gentamicin: significant worsening of high-frequency (4000 - 8000 Hz) thresholds (59.05 ± 17.4 → 69.4 ± 21.98 dB, p<0.01) and caloric weakness (45.8% → 71.5%, p<0.01). Selective window method using gel foam soaked in dexamethasone: no significant change in PTA (low or high frequencies), but caloric weakness increased (37.6% → 54.6%, p<0.05).	The results of this study may suggest that dexamathasone has a protective effect for gentamicin toxicity.	Small number of study participants; retrospective design; non-randomized (patients self-selected method); lack of blinding. The primary aim of this study was not to determine whether dexamethasone has protective properties against gentamicin-induced ototoxicity, but rather to compare two treatment approaches for

								Ménière's disease.
